# Mood disorder with mixed, psychotic features due to vitamin b12 deficiency in an adolescent: case report

**DOI:** 10.1186/1753-2000-6-25

**Published:** 2012-06-22

**Authors:** Ali Evren Tufan, Rabia Bilici, Genco Usta, Ayten Erdoğan

**Affiliations:** 1 Assistant Professor, Abant Izzet Baysal University Medical Faculty, Department of Child and Adolescent Psychiatry, Baysal, Turkey; 2 Psychiatrist, Elazig Hospital for Mental Disorders, Elazig, Turkey; 3 Child and Adolescent Psychiatrist, Private Practice, Ankara, Turkey; 4 Associate Professor, Bakirkoy Prof. Dr. Mazhar Osman Hospital for Mental Health and Nervous Disorders, Department of Child and Adolescent Psychiatry, Osman, Turkey; 5 Abant Izzet Baysal Universitesi, Tip Fakültesi, Cocuk ve Ergen Psikiyatri Anabilim Dali, 14280, Golkoy, Bolu, Turkey

**Keywords:** Vitamin B12, psychosis, mood disorder, extrapyramidal symptoms

## Abstract

Vitamin B12 is one of the essential vitamins affecting various systems of the body. Reports of psychiatric disorders due to its deficiency mostly focus on middle aged and elderly patients. Here we report a case of vitamin B 12 deficiency in a 16-year old, male adolescent who presented with mixed mood disorder symptoms with psychotic features. Chief complaints were “irritability, regressive behavior, apathy, crying and truancy” which lasted for a year. Premorbid personality was unremarkable with no substance use/exposure or infections. No stressors were present. The patient was not vegetarian. Past medical history and family history was normal. Neurological examination revealed glossitis, ataxia, rigidity in both shoulders, cog-wheel rigidity in the left elbow, bilateral problems of coordination in cerebellar examination, reduced swinging of the arms and masked face. Romberg’s sign was present. Laboratory evaluations were normal. Endoscopy and biopsy revealed atrophy of the gastric mucosa with Helicobacter Pylori colonization. Schilling test was suggestive of malabsorbtion. He was diagnosed with Mood disorder with Mixed, Psychotic Features due to Vitamin B12 Deficiency and risperidone 0.5 mg/day and intramuscular vitamin B12 500 mcg/day were started along with referral for treatment of *Helicobacter pylori*. A visit on the second week revealed no psychotic features. Romberg’s sign was negative and cerebellar tests were normal. Extrapyramidal symptoms were reduced while Vitamin B12 levels were elevated. Risperidone was stopped and parenteral Vitamin B12 treatment was continued with monthly injections for 3 months. Follow-up endoscopy and biopsy at the first month demonstrated eradication of H. pylori. He was followed monthly for another 6 months and psychiatric symptoms did not recur at the time of last evaluation. Despite limitations, this case may underline the observation that mood disorders with psychotic features especially with accompanying extrapyramidal symptoms lacking a clear etiology may be rare manifestation of vitamin B12 and/or folate deficiency in children and adolescents and be potentially amenable to treatment.

## Background

Vitamin B12 is one of the essential vitamins affecting various systems of the body. In case of deficiency; hematologic (megaloblastic, macrocytic anemia), neurologic (demyelinization, paresthesia), gastrointestinal (anorexia, glossitis) as well as psychiatric symptoms arise. The psychiatric symptoms may not be concurrent with symptoms arising from other systems and even may precede them [1-3]. The symptoms may include agitation, irritability, negativism, confusion, disorientation, amnesia, impaired concentration and attention and insomnia; while psychiatric disorders that may be diagnosed in patients having vitamin B12 deficiency include depression, bipolar disorder, panic disorder, psychosis, phobias and dementia [1-6]. In adult patients, the clinical picture may especially involve affective or psychotic symptoms. These observations may be explained by the importance of vitamin B 12, folate and homocysteine in carbon transfer metabolism (methylation) required for the production of serotonin, other monoamine neurotransmitters and catecholamines [1-6]. Up to now, reports of psychiatric disorders due to vitamin B12 deficiency mostly focused on middle aged and elderly patients and pediatric cases are reported to be rare [7-9]. This study aimed to report a case of vitamin B 12 deficiency in an adolescent who presented with mixed mood disorder symptoms with psychotic features.

### Case presentation

A sixteen-year old male adolescent was brought to our department with complaints of “irritability, regressive behavior, apathy, crying and truancy”. Upon questioning of his parents it was learned that the complaints have been present for the past year. The patient had started to display anxiety during separation from his mother, wept frequently and complained of vague pains, lethargy, forgetfulness and reduced concentration alternating with racing thoughts, irritability, anhedonia, distractibility, reduced sleep and appetite. Speech was reduced and he became progressively isolated from his peers. He refused to go to school and when sent became truant frequently. His parents reported that he was frequently agitated, spent his free time in front of his computer and that he ran excessive debts on their credit cards buying items on-line. There was no previous history of compulsive shopping or buying sprees. Premorbid personality of the adolescent was described as extrovert, euthymic and active. He was well liked by his friends although the academic staff reported problems in attention starting from the second grade. His teachers also reported fidgetiness and impulsivity which was especially prominent in mathematics lessons.

In the mental status examination, impaired attention, concentration, as well as insomnia, reduced working and short-term memory, elementary auditory (i.e. knocking and ringing), olfactory (i.e. burnt rubber, perfumes and tobacco), and visual hallucinations (i.e. a white, man-like shape, especially present in the evenings), as well as passive suicidal ideation were noted. Judgment, abstract thought and reality testing were impaired. Speech was hypo-phonic, thought process was sluggish. Thought content was found to be impoverished and dominated by somatic complaints, delusions of reference, guilt and thought broadcasting. Mood was blunted and the affect was restricted in range. Psychomotor activity and appetite were reduced.

The patient reported that those complaints arose in the past year and that hallucinations were added in the past three months. Within the last month, delusions of reference (i.e. thinking that others were looking at and talking about him), delusions of guilt (i.e. that he had sinned and would be punished) arose and the patient reported that his thoughts were broadcast so that others can read and understand his thoughts. No history of psychoactive substance use, encephalitis, use of antipsychotics/antiemetics, exposure to carbon-monoxide or organophosphate compounds or stressors was present. The patient was not vegetarian. Past medical history and family history was unremarkable for both psychopathology and chronic medical disorders. Physical and neurological examination revealed glossitis, ataxia, rigidity in both shoulders, cog-wheel rigidity in the left elbow, bilateral problems of coordination in cerebellar examination, reduced swinging of the arms and masked face. Romberg’s sign was present although no signs or symptoms of peripheral neuropathy could be observed. Evaluation with The Extrapyramidal Symptom Rating Scale (ESRS) yielded a score of 19, with items of bradykinesia and parkinsonism being positive [10].

Electroencephalography, electromyography, somatosensorial evoked potentials, cerebrospinal fluid analysis, cranial MRI, thyroid and liver function tests, pancreatic enzymes, electrolytes, parathormone, ceruloplasmin and whole blood count were within normal limits. HIV (ELISA) was negative. Twenty-four hour urine copper level was within normal ranges. An ophthalmologic examination ruled out the presence of a Kayser Fleischer ring. A peripheral blood smear with Wright’s stain was found to be normal. Endoscopy revealed atrophy of the gastric mucosa while a biopsy sample taken during endoscopy revealed colonization with Helicobacter Pylori. A Schilling test was administered to determine the etiology of Vitamin B12 deficiency and it was observed that radiolabeled Vitamin B12 levels were low both for Stage I and II of the test which was thought to reflect malabsorbtion [11].

Psychometric testing with the Beck Depression (BDI) and Anxiety Inventories (BAI) revealed scores of 35 (Cut-off score = 17, Severe Depressive Symptoms) and 36 (Without a clinically defined cut-off score albeit denoting severe anxiety), respectively [12,13]. An evaluation with the Turkish version of the Young Mania Rating Scale (YMRS), which does not have clinically designated cut-off score, yielded a score of 13 [14]. Psychotic symptoms were evaluated with Positive and Negative Syndrome Scale and the patient scored 20, 23 and 56 for the Positive, Negative and General Psychopathology subscales (Total 99) [15]. Vitamin B 12 levels were found to be 166 ng/mL in two subsequent tests after 6–8 hours of fasting with immunoassay method via Advia Centaur XT^TM^ (Normal 197–400 ng/mL) while folate and transcobalamine levels were normal. Hemoglobin was found to be 10 g/dL (Normal 14–18 g/dL) and MCV was 98 fL (Normal 80- 100 fL) [16]. Bone marrow examination did not reveal megaloblastic changes.

As a result of the history and evaluations and noting that the psychotic symptoms were superimposed on affective-anxious symptoms, the patient was diagnosed as having Mood disorder with Mixed, Psychotic Features due to Vitamin B12 Deficiency according to DSM-IV-TR criteria and risperidone 0.5 mg/day and intramuscular vitamin B12 500 mcg/day were started for treatment [17]. Risperidone was chosen because of it being one of the most commonly used atypical antipsychotics for management of psychosis, pervasive developmental disorders, mental retardation, mood disorders and disruptive behavior disorders in children and adolescents and having no known interaction with vitamin B12. The usual dose range of risperidone for acute psychosis and mood disorder is reported to be 2–8 mg/day while we have started risperidon at 0.5 mg/day to help stabilize the patient while the medical work-up and treatments were being completed [18]. Vitamin B12 was the only vitamin supplement started.

**Figure 1:**
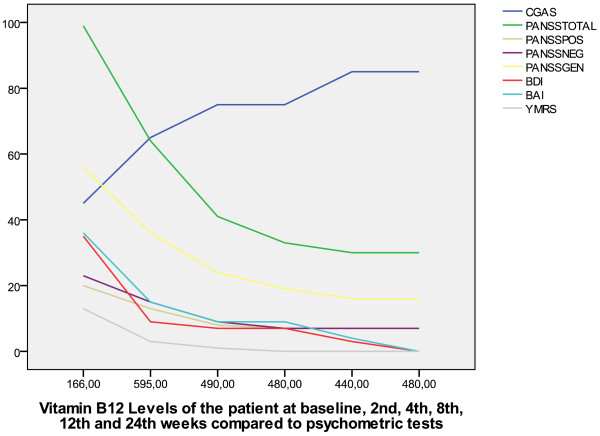
Vitamin B12 levels of the patient at baseline, 2^nd^, 4^th^, 8^th^, 12^th^ and 24^th^ weeks compared to changes in psychometric tests.

At the same time the patient was referred for treatment of *Helicobacter pylori* and was prescribed clarithromycin 1000 mg/day, lansoprazole 60 mg/day and amoxicillin 2000 mg/day. A follow-up visit on the second week revealed that no psychotic features were present, Romberg’s sign was negative and that cerebellar tests were within normal limits. Extrapyramidal symptoms were reduced. Both the patient and his mother reported that apathy, crying, regressive behavior and truancy were reduced. An evaluation with BDI and BAI revealed scores of 9 and 15, respectively while Evaluation with ESRS and YMRS yielded a score of 3 for both. PANSS scores for positive, negative and general psychopathology subscales were 13, 15 and 36 respectively (Total 64). Vitamin B12 levels were measured at this visit as 595 ng/mL. At the scond week, risperidone was stopped and parenteral Vitamin B12 treatment was continued with monthly injections for 3 months. The time course of changes in BDI, BAI, PANSS and its subscales, YMRS and ESRS according to Vitamin B12 levels is illustrated in Figure 1.

A follow-up endoscopy and biopsy at the first month revealed that *H. pylori* were eradicated from the gastric mucosa. The patient was followed for another 6 months at monthly intervals and the psychiatric symptoms did not recur at the time of last evaluation.

This study reports a case of vitamin B12 deficiency in an adolescent who presented with mixed mood disorder symptoms with psychotic features as well as extra-pyramidal symptoms and its response to Vitamin B12 treatment. Although concurrent administration of risperidone in our patient may be considered as a limitation in light of reports of improvement in psychotic symptoms as early as the first week, the fact that the administered dose of 0.5 mg/day was lower than doses recommended for controlling acute psychosis and mood disorders may support the beneficial effect of treatment with Vitamin B12 [18]. The course of improvement in psychometric measures with changes in Vitamin B12 levels may also support this hypothesis (Figure 1).

Up to now, there was only one case report of psychotic disorder, again concomitant with extrapyramidal symptoms associated with Vitamin B12 and folate deficiency in a 12-year old male adolescent and this case was also reported from Turkey [8]. The time course and response to treatment as well as the content of the symptoms of the patient reported by Dogan and colleagues [8] seems to be similar to our patient and although the folate levels in our patient were within normal limits it may be argued that vitamin B12 deficiency may have masked concurrent folate deficiency in our patient.

It is also unclear why Vitamin B12 deficiency leads to prominent neuro-psychological symptoms in some patients and not others. C677T polymorphism of the methylene-tetrahydro folate reductase (MTHFR) gene, which has an elevated prevalence in Mediterranean countries, may protect patients defective for Vitamin B12 levels against the emergence of hematological symptoms and homozygosity for this gene may be elevated in Turkish populations, especially from Eastern Turkey [1,8,19].

Another interesting feature of both our patient and that reported by Dogan and colleagues seem to be the prominence of extra-pyramidal symptoms in presentation [8]. This may be due to the dissociation between neurological and hematological symptoms, presumably due to MTHFR polymorphism or alternatively it may be due to changes in basal ganglia, especially globus pallidus as observed in Methly-malonic academia [20]. It may also be posited that patients with less severe mutations of methyl-malonyl coA mutase may be asymptomatic initially and reduced activity of the enzyme, perhaps in situations of vitamin B12 deficiency may lead to changes in basal ganglia [20]. It may also be striking that both our patient and the other, two cases of vitamin B12 deficiency with extra-pyramidal symptoms are males [8,20]. Although the lack of evaluation of volumes of basal ganglia in our patient may also be a limitation of our study, it may be said that future studies evaluating volumes of basal ganglia in patients with Vitamin B12 deficiency are needed.

The pathophysiology of extrapyramidal symptoms in patients with Vitamin B12 deficiency is far from clear, although some indirect evidence exists. Firstly, S-Adenosylmethionine (SAM) in carbon transfer metabolism is the only methyl donor in the Central Nervous System and knockout mice for the MTHFR enzyme have been reported to have reduced SAM, global DNA hypomethlyation and cerebellar pathology [21]. Secondly, cysteine which is a precursor for the major intracellular redox buffer glutathione is a breakdown product of homocysteine and therefore the carbon transfer metabolism. Neurons lack this pathway and are dependent on glia for production, thereby rendering them more susceptible to oxidative damage [1]. The facts that MDA (malondialdehyde) is a measure of membrane lipid peroxidation and that MMA (methyl malonic academia) is an inborn error of metabolism usually presenting with acute extrapyramidal symptoms in infants sometimes responds to vitamin B12 treatment also support the importance of vitamin B12 in preventing oxidative damage, perhaps especially for dopaminergic neurons [1,8,22]. Thirdly, MTHFR is also involved in the metabolism of tetrahydrobiopterin and the latter is required for synthesis of dopamine and serotonin. It also acts directly on specific membrane receptors to release monoamine neurotransmitters as well as having specific protective antioxidant effects on dopamine neurons [1]. Dopamine also stimulates methylation of phospholipids in the neuronal membrane and this reaction depends on single carbon folate pathway, thereby underlining the importance of the relationship between dopaminergic neurotransmission and single carbon metabolism [23]. Lastly, the cholinergic synapses may also be involved in the pathophysiology because of the dependence of choline synthesis on SAM [1]. Globus pallidus among the basal ganglia may be expecially vulnerable to these pathophysiological mechanisms [20]. Regardless of the pathophysiological mechanism involved, further studies involving larger samples are needed to determine the prevalence of extrapyramidal symptoms in vitamin B 12 deficient patients from varying age cohorts.

## Conclusions

This study reports a case of vitamin B12 deficiency in an adolescent male presenting with mood disorder with mixed features. The lack of evaluation of volumes of specific brain structures, concurrent administration of an atypical antipsychotic along with vitamin B12, further evaluation of small intestine flora to determine the causes of bacterial over growth other than *H. pylori*, evaluation of RBC folate levels along with levels in serum of patient, evaluation of Homocysteine levels and testing for mutations in MTHFR and methyl-malonyl coA mutase, celiac antibodies and anti-parietal cell titres may be counted among the limitations of the case presentation. Also, we could neither explain the reduction in Hb in absence of bone marrow RBC precursor megaloblastosis nor the rarity of this presentation in face of prevelance of mutations in MTHFR/borderline low Hb and Vitamin B12 levels or the presence of Romberg Sign along with extrapyramidal symptoms and therefore the observed presentation may be deemed spurious.

Regardless of its limitations, our case may underline the observation that mood disorders with psychotic features especially with accompanying extrapyramidal symptoms lacking a clear etiology may be rare manifestation of vitamin B12 and/or folate deficiency in children and adolescents and be potentially amenable to treatment. Evaluation of Vitamin B12 and folate levels may be prudent in children and adolescents with affective complaints of abrupt onset and with accompanying psychotic/motor features.

### Consent

Written informed consent was obtained from the patient for publication of this Case Report and any accompanying images. A copy of the written consent is available for review by the Editor-in-Chief of this journal.

## Abbreviations

ESRS, Extrapyramidal Symptom Rating Scale; BDI, Beck Depression Inventory; BAI, Beck Anxiety Inventory; YMRS, Young Mania Rating Scale; MTHFR, methylene-tetrahydro folate reductase; SAM, S-Adenosyl methionine; MDA, malondialdehyde; MMA, methyl malonic academia.

## Competing interests

The author(s) declare that they have no competing interests.

## Authors’ contributions

AET and RB diagnosed the patient, provided treatment and follow-up, GU helped write and prepare manuscript and AE supervised manuscript preparation and evaluated final draft, providing suggestions for discussion. No part of this study has been submitted elsewhere for publication and the authors have no financial affiliation or supports to disclose. All efforts have been undertaken to maintain the ethical standards in the study and informed consent was procured from the parents as well as the assent of the adolescent concerned. This case was presented as a poster at the 14^th^ Adolescence Days (10^th^ – 12^th^ December 2009, Ankara, TURKEY). All of the authors have made genuine and substantial contributions to this paper, read it in detail and approved its release.
